# Centration in refractive surgery

**DOI:** 10.5935/0004-2749.20200014

**Published:** 2020

**Authors:** Lívia Cristina Rios, Patrícia Gomes da Silva, Aristófanes Mendonça Canamary Junior, Pablo Rodrigues, Caio Vinícius Saito Regatieri, Mauro Campos, Bernardo Kaplan Moscovici

**Affiliations:** 1 Unidade Paulista de Oftalmologia, São Bernardo do Campo, SP, Brazil; 2 Universidade Federal de São Paulo, São Paulo, SP, Brazil

**Keywords:** Refractive surgical procedures/methods, Cornea/ pathology, Pupil/physiology, Ocular fixation, Cornea/surgery, Excimer lasers, Procedimentos cirúrgicos refrativos/métodos, Cór nea/patologia, Pupila/fisiologia, Fixação ocular, Córnea/ ci rur gia, Lasers de excimer

## Abstract

The point of centration for refractive surgery is a theme of great importance that generates considerable discussion among specialists and surgeons in the field. Notably, any changes in light can alter the size of the pupil, and the visual axis of the fixation line to the fovea is unique in each patient. A variety of options have been described in the literature with respect to centration in refractive surgery, and the results differ among these methods. No consensus has been established regarding the ideal refractive surgery technique for evaluation of centration in each patient that will yield a satisfactory surgical result.

## Abstract (pt)

O ponto de centralização da cirurgia refrativa é tema de grande importância e gera muita discussão entre especialistas e cirurgiões da área. Afinal, qualquer alteração na luz pode alterar o tamanho da pupila, além disso, o eixo visual da linha de fixação para a fóvea é particular em cada paciente. Existem opções para centralização em cirurgia refrativa com resultados diferentes na literatura. Ainda não há consenso sobre a melhor técnica em cirurgia refrativa que avalie cada caso específico visando um resultado cirúrgico final satisfatório.

## INTRODUCTION

The eye is not a perfect optical system. If it were, it would include a visual axis linking the object of fixation directly to the foveola, passing through the nodal point of the eye and the optical centers of all ocular elements. Because no such system exists, when centering glasses, contact lenses, and refractive procedures, some reference landmarks must be used^(1^-^2)^. The two most important points of reference for centration are the pupil center (PC) and the corneal vertex (CV). The CV is defined by the closest point to the center of the Placido image on corneal topography; thus, it is very close to the Purkinje reflex, but almost never coincident with the geometric center of the cornea. The PC is represented by the classical image of the pupil through the cornea, and is important because the iris acts as a natural light barrier. The importance of the PC is demonstrated by the Stiles-Crawford effect, in which the principal ray of light entering the eye passes through the PC. Thus, the PC represents the point with the greatest luminosity or highest amount of light^(1^,^3^,^4)^.

**Figure 1:**
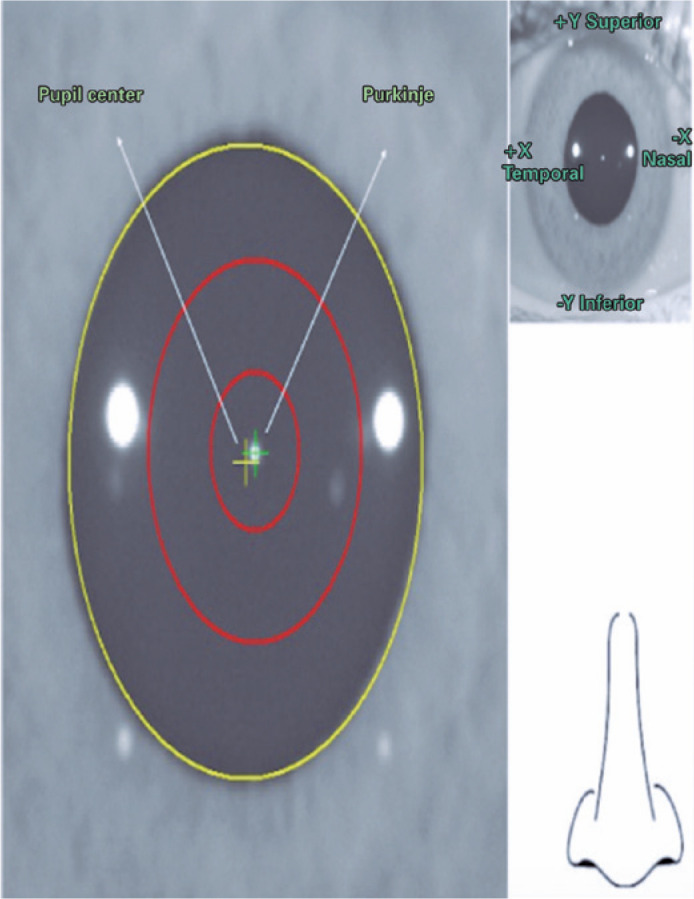
Corneal Vertex and Pupil Center using AcuTarget (SensoMotoric Instruments, Teltow, Germany).

Another important concept is the (imaginary) line of sight, which links the object of fixation to the macula. The visual center is where this line passes through the cornea^(1^-^2)^. The pupillary axis is a line perpendicular to the cornea, which passes through the center of the pupil. Because the visual axis cannot be used as a reference, the line of sight that joins the object of fixation to the PC is used in its place^(1^-^2)^ (Figure 1). The lambda angle, originally known as the kappa angle (in theory, the two concepts are distinct, but there is no clinical difference) is an important parameter to consider. This angle occurs when patients direct their gaze toward a luminous punctate object of fixation^(1^-^3)^. However, the reflection of that object is far from the center of the entrance pupil, simulating strabismus but eliciting physiological ocular alignment. The kappa angle was thus originally defined as the angular distance between the visual and pupillary axes. However, as the visual axis is purely theo retical, the kappa angle was redefined as the angular distance between the line of sight (corneal apex) and the pupillary axis^(1^-^3)^.

**Figure 2:**
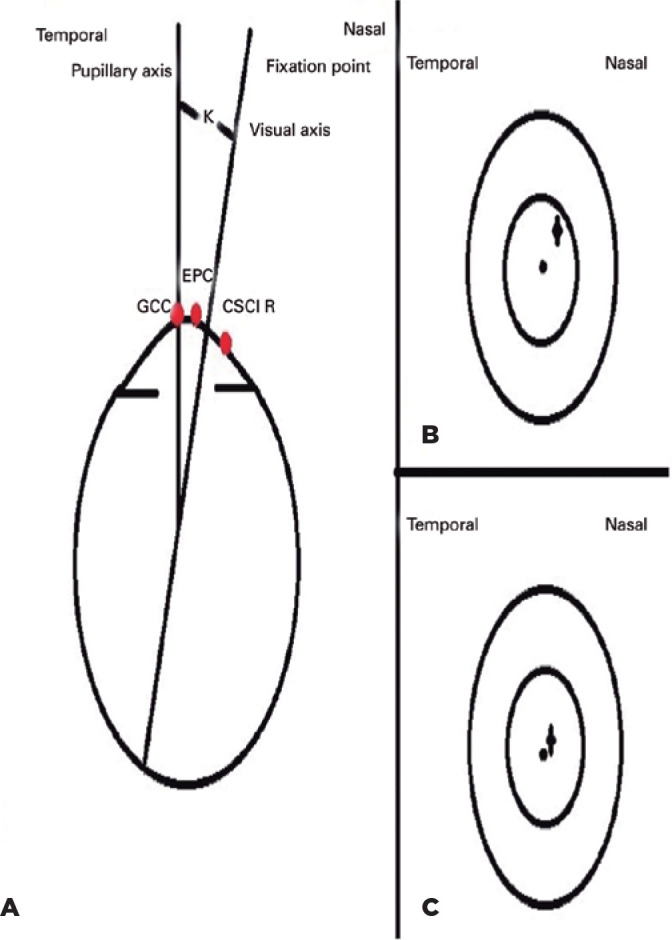
Geometric center of the cornea (GCC), entrance pupil center (EPC), and coaxially sighted corneal light reflex (CSCLR) as identified by Pande and Hillman,^[^21^]^ (b) Surgeon’s view of a large angle kappa, (c) Surgeon’s view of a normal but small positive angle kappa. Reference: Moshirfar M, Hoggan RN, Muthappan V. Angle Kappa and its importance in refractive surgery. Oman J Ophthalmol. 2013;6(3):151-8. ^(15)^

**Figure 3:**
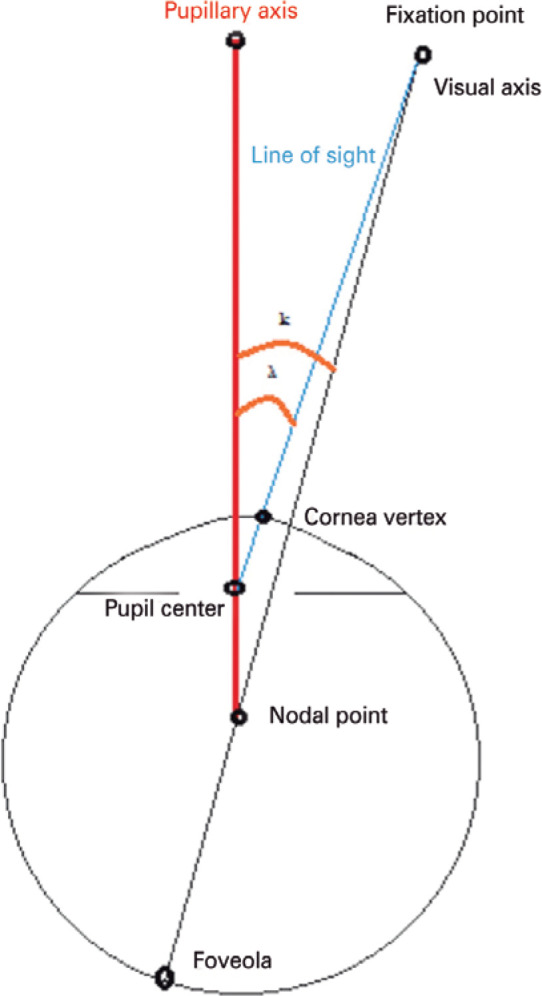
Schematic drawing of reference points: corneal vertex; pupil center; pupillary axis; visual axis; and line of sight.

The Purkinje (or Purkinje-Sanson) reflexes are the reflections of objects in the structure of the eye, which can form four distinct images. The most important image in clinical practice is the first image: the reflection of the outer surface of the cornea (superior nasal) (Figures 2 and 3). The second image is the reflection of the inner surface of the cornea, while the reflections of the outer (anterior) and inner (posterior) surfaces of the lens constitute the third and fourth images, respectively. Thus, when gaze is fixed on a point of light, the Purkinje reflex is typically not centered; it may be nasal (positive kappa angle) or temporal to the pupillary center (negative kappa angle). The distance from CV to PC, typically measured with Scheimpflug devices, can be used to estimate this angle^(1^-^3)^. Furthermore, the coaxially sighted corneal light reflex axis could be used as a centration point, as it can be obtained directly from the surgical microscope when the other eye is closed.

(o)= EPC (+)= CSCLR.

**Figure 4:**
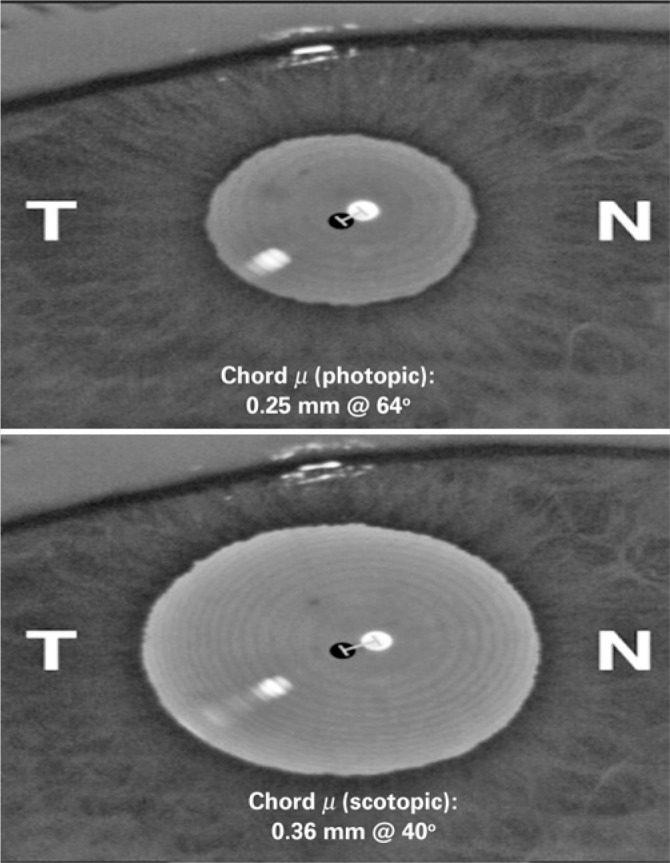
Chord mu (m) as measured under (top panel) photopic and (bottom panel) scotopic light conditions. While the position of subject-fixated coaxially sighted corneal light reflex does not change under different lighting conditions, the center of the entrance pupil is altered with dilation, resulting in an increased chord m under scotopic conditions.

A two-dimensional reference point for use as a clinical reference would be of great value: chord mu (µ) (Figure 4). Chord mu represents the displacement of the entrance PC from the subject-fixated coaxially sighted corneal light reflex. Although it references the distance between two points on a given plane, rather than the angles between two lines, it changes as the frame of reference moves from the lens-iris plane to the corneal plane. In clinical practice, the change in chord mu between the lens-intraocular lens (IOL) plane and the corneal plane is typically not significant.

Reference: CHANG DH; WARING GO 4th. The subject-fixated coaxially sighted corneal light reflex: A clinical marker for centration of refractive treatments and devices. Am J Ophthalmol 2014;158:863-874.

T= temporal; N= nasal.

## DISCUSSION

Given this variety of definitions, the utility of the aforementioned principles may be unclear. In most cases, the distance between CV and PC is very small, whereas in others, especially in eyes with high refractive error and some conditions such as keratoconus, larger kappa angles may be found. In any refractive procedure, as well as when placing corrective and intraocular lenses, centration has a major influence on daily practice. In the past, the most commonly reference point used was the PC, because it represents the center of the light that passes through the lens directly to the retina. However, most practitioners believe that an imaginary line passing through the PC to the macula would yield better results because of the Stiles-Crawford phenomenon. An alternative perspective is that the CV represents the closest point to the imaginary line of vision and the Purkinje reflex, and would thus yield better results^(1^-^3)^.

Refractive surgery with excimer laser generally has satisfactory results. However, controversy remains with respect to whether the ablation should be centered on the PC or CV. Most excimer laser devices are equipped with tracking functionality, which uses the PC as a landmark, rather than the CV. Most eye tracker devices in excimer lasers are ultrafast, high-latency, multi-dimensional, and use different sources of light; some use infrared light to provide better results. Other improvements of excimer laser devices include cyclotorsion correction, as well as iris or limbal recognition that can improve the refractive correction of astigmatism; however, no improvements in centration accuracy have been observed. Some studies are underway to determine which centration point produces the best results^(5)^.

The optimal mathematical model for centration remains unknown, but the search continues. In another study, Arba Mosquera and Ewring^(6)^ used the CV (measured by videokeratoscopy) as a landmark to center refractive procedures on the cornea, while Uozato and Guyton^(7)^ recommended the PC as a reference for refractive surgeries. De Ortueta and Schereyger^(2)^ used the CV as a centration landmark and obtained good results in refractive photoablation. Soler et al.^(8)^ found better results in hyperopic patients with increased kappa angles by using PC-based centration, although most other authors consider these patients ideal for CV-based centration. In the present study, a smaller amount of induced coma and improved best-corrected visual acuity were found. Clinically, the coma value is not as important as the measurement of high-order aberrations, if performed on the basis of PC. Therefore, if treatment is centered on the pupil, aberration rates will be lower, although this may not correlate with patient satisfaction^(2^,^6^-^9)^.

Reinstein et al.^(10)^ reported a case in which a better treatment profile was achieved with CV centration after radial keratotomy. In another study^(3)^, the same authors could not find differences in complaints of contrast sensitivity and visual acuity in hyperopic patients who had kappa angles smaller than 0.55 mm and were treated with CV centration. In addition, no differences were found in groups with kappa angles up to 0.25 mm and up to 0.55 mm. Reinstein et al. found greater induction of high-order aberrations (mainly coma) when CV centration was used, potentially as a result of the factors mentioned previously. However, the most important observation in that study was that patients did not report night-vision complaints or any postoperative alterations in contrast sensitivity; according to those authors, these results suggest that patients see through the CV, and that no visual acuity is lost when centration is done at this point^(3^,^11)^.

Arbalaez et al.^(12)^ reported better corrected visual acuity in myopic patients with high kappa angles, as well as better sphericity indices and better postoperative aberrometry results, with CV centration. In a study of multifocal lens implants in dissatisfied patients, Prakash et al.^(13)^ found that many patients had increased kappa angles. Chan and Boxer Wachler^(14)^ used the Purkinje reflex for centration and achieved better results than when using the PC of the contralateral eye^(2^,^13^,^15)^ (Table 2). Soler et al.^(8)^ showed results with larger coma with PC in eyes with small kappa angle and with CV in eyes with large kappa angle (Table 1). Gatinel et al. ^(16)^ showed better results when centering corneal inlays on CV^(16)^.

**Table 1:** Literature review of paper with different centration method:

	Preoperative SE	Ablation center	Excimer used	Results
Chan et al.^(14)^	+1.875	Purkinje	VISX 52	UCVA, better refractive results when compared with the contralateral eye, PC-centered
Nepomuceno et al.^(20)^	+2.73±1.41	Purkinje	LadarVision 4000	MRSE: 0.25±0.82 (D)/UCVA: 44.4% 20/20 and 81.5% 20/30 or better
Chang et al.^(4)^	+2.17±0.93	Purkinje	LadarVision 4000	UCVA (logMAR): 0.22±0.17
Kermani et al.^(24)^	+2.57±1.56 (VA)	VA and PC	NIDEK MK 2000	MRSE: +0.29±0.70 D (VA) vs. +0.19±0.57 D (PC). 81% of eyes (VA) with less than ±0.50 D vs. 64% (PC). Less coma
	+2.46±1.32 (PC)			induction in the VA group
De Ortueta et al.^(2)^	+2.76±0.90	CV	Esiris	MRSE: +0.09±0.32 D. 94% eyes with less than ±0.50 D
Soler et al.^(8)^	+2.69±0.91 (VC)	CV and PC	Allegretto 200 Hz	Similar refractive results. In terms of coma in eyes with SAK:
	+2.26±0.62 (PC)			0.60±0.24 µm (PC) vs. 0.34±0.30 µm (CV). In eyes with LAK: 0.34±0.30 µm (PC) vs. 0.62±0.67 µm (CV)
Reinstein et al.^(10)^	+3.85±0.98 (SAK)	Purkinje	MEL 80	Similar refractive results, nearly equal contrast sensitivity, increase
	+3.87±0.90 (LAK)			in total ocular aberrations in patients with LAK, mainly coma

SE= spherical equivalent; SAK= small angle kappa; LAK= large angle kappa; VA= midpoint between reflex; D, diopter; UCVA= uncorrected visual acuity; logMAR= logarithm of the minimum angle of resolution; MRSE= manifest refraction spherical equivalent; PC= pupil center; CV= corneal vertex; µm= micrometers; vs= versus.

**Table 2:** Results of study by Arbalez et al. at 6-month follow-up, based on centration

	CV group	PC group
Defocus (D)	-0.26 ± 0.49	-0.29 ± 0.40
Astigmatism (D)	0.15 ± 0.18	0.19 ± 0.21
BSCVA	1.24 ± 0.47	1.20 ± 0.45
Coma (µm)	0.23 ± 0.11	0.27 ± 0.17
Trefoil (µm)	0.15 ± 0.09	0.15 ± 0.08
Spherical aberration (µm)	0.09 ± 0.29	0.14 ± 0.30
High-order RMS (µm)	0.48 ± 0.12	0.51 ± 0.17
Pupillary offset (mm) (considering 6-mm pupil)	0.28 ± 0.11	0.31 ± 0.19

Purkinje and PC. CV= corneal vertex; PC= pupil center; RMS= root mean square; BSCVA= best spectacle-corrected visual acuity.

Following this review of the literature, the next step is to evaluate the information received by the devices. Corneal topography measurements are not centered over the PC, but rather over the first Purkinje image. To the best of our knowledge, the Pentacam (Oculus, Wetzler, Germany) calculates the kappa angle in the same manner, potentially by using the point of highest elevation as the CV. Other devices, such as OPD-Scan III (NIDEK, Inc., Fremont, CA, USA), AcuTarget (SensoMotoric Instruments, Teltow, Germany), and Galilei (Ziemer Ophthalmic Systems, Port, Switzerland) may use a similar approach. Notably, the iTrace device (Tracey Technologies, Houston, TX, USA) uses other angles, such as the alpha angle (defined as the distance from normal vertex to the center of white to white). However, this does not actually correspond to the alpha angle-the correct definition is the angle between the optical axis of the eye and the visual axis-and is therefore an approximation.

Our review of the literature found increasing evidence of the importance of the kappa angle in centration of refractive procedures, especially in eyes with high angles and high refractive errors. Excimer laser centration is more difficult and less accurate when using the Purkinje reflex. Some excimer devices have the option to program the treatment center using Scheimpflug data, based on the distance between PC and CV. Some surgeons presume that, when this difference is greater than 0.3 mm and the kappa angle is greater than 0.5 mm, the results will be better with CV centration; however, there is no real evidence of this and no cutoff value in terms of the kappa angle that indicates a preference for CV or PC^(1^,^15)^. The choice to center on the CV is especially frequent in cases of high hyperopia and astigmatism. Some studies have shown the potential for asymmetric centration on one-half or three-quarters of the distance between the CV and PC^(4^,^16^,^17)^.

Another important aspect of the kappa angle in daily practice is preoperative topographic evaluation. In eyes with a high kappa angle, the topographic image may not be the central image, but may be displaced to the periphery. This can be demonstrated by similar and corresponding regions, with non-standard flattening expected at the periphery of the axial map^(18^,^19)^. Current refractive surgery devices allow millimeter-level shifts in centering, away from the pupillary center. Allegretto EX500 software (Alcon Labs, Fort Worth, TX, USA) allows the operator to position the centering at 25%, 50%, or 75% of the distance between the CV and PC. In aberrometry-guided surgeries, most aberrometers use the PC, except iDesign (Abbott Laboratories, Abbott Park, IL, USA), which uses the center of the cornea. The Schwind Amaris 1050RS (Eye Tech Solutions, Kleionstheim, Germany) and Mel 90 (Carl Zeiss Meditec, Jena, Germany) devices allow manual changes in treatment centration, but topography-guided treatments are centered on the CV and wave-guided treatments are centered on the PC^(20^,^21)^. One notable example of centralized laser techno logy is applicable to lasers (Table 3).

**Table 3:** Summary of centration techniques applied by various commercial laser refractive systems

	Device	Technique	Applied	Type
WaveLight	Allegretto Allegretto-Eye-Q EX500 Concept 1000	Manually based CLR (but not truly CS), for large offsets or angles (alpha, kappa, lambda) “in between”	Under the laser	Overall ablation is shifted
SCHWIND	AMARIS AMARIS 500E AMARIS 750S AMARIS 1050RS	Manually based on corneal vertex (numerically taken from diagnosis)	During treatment planning	The optical axis is shifted (even for customized treatments), but the overall ablation remains concentric to the pupil boundaries
ZEISS Meditec	MEL80 MEL90	Manually based CLR (but not truly CS), considering contralateral viewing eye to reduce parallax	Under the laser	Overall ablation is shifted
Nidek	Quest	Manually based CLR (but not truly CS)	Under the laser	Overall ablation is shifted
Bausch & Lomb 217 Zyoptix	217 Zyoptix	Manually based CLR (but not truly CS)	Under the laser	Overall ablation is shifted
Novatec	LightBlade	Manually based CLR (but not truly CS)	Under the laser	Overall ablation is shifted

CLR= corneal light reflex; CS= coaxially sighted.

The precision of new excimer laser devices and new intraocular lenses demands an increasing degree of excellence in their use. Ideal centration is an important factor in optimizing surgical results^(22^-^24)^. Most studies have shown that the kappa angle directly influences refractive procedures; however, further research is needed to more clearly elucidate this relationship. Clinical trials with larger numbers of patients should provide more information, as advances in excimer lasers and wider optical-zone treatments have caused difficulty in establishing statistical significance. In addition, it may be useful to determine the point through which each patient fixates on objects.
